# The Spatial-Temporal Characteristics and Influential Factors of NOx Emissions in China: A Spatial Econometric Analysis

**DOI:** 10.3390/ijerph15071405

**Published:** 2018-07-04

**Authors:** Beidi Diao, Lei Ding, Panda Su, Jinhua Cheng

**Affiliations:** 1 School of Economics and Management, China University of Geosciences, 388 Lumo Road, Wuhan 430074, China; beididiao@cug.edu.cn; 2 School of International Business & Languages, Ningbo Polytechnic, 1069 Xinda Road, Ningbo 315800, China; 3 School of Public Administration, China University of Geosciences, 388 Lumo Road, Wuhan 430074, China; supanda@live.cn

**Keywords:** NOx emissions, STIRPAT, socioeconomic influential factors, spatial correlation analysis, spatial econometric models

## Abstract

While the progress of China’s industrialization and urbanization has made great strides, atmospheric pollution has become the norm, with a wide range of influence and difficult governance. While many previous works on NOx pollution have been developed from the perspectives of natural science and technology, few studies have been conducted from social-economic points of view, and regional differences have not been given adequate attention in driving force models. This paper adopts China’s provincial panel data from 2006 to 2015, an extended STIRPAT (Stochastic Impacts by Regression on Population, Affluence and Technology) model, and spatial econometric models to investigate the socio-economic influential factors and spatial-temporal patterns of NOx emissions. According to the spatial correlation analysis results, the provincial NOx emission changes not only affected the provinces themselves, but also neighboring regions. Spatial econometric analysis shows that the spatial effect largely contributes to NOx emissions. The other explanatory variables all have positive impacts on NOx emissions, except for the vehicular indicator (which did not pass the significance test). As shown through the estimated consequences of direct and indirect effects, the indicators have significant positive effects on their own areas, and exacerbate NOx pollution. In terms of indirect effects, only three factors passed the significant test. An increase in gross domestic product (GDP) and energy consumption will exacerbate adjacent NOx pollution. Finally, a series of socio-economic measures and regional cooperation policies should be applied to improve the current air environment in China.

## 1. Introduction

In recent years, due to the progress in China’s industrialization and urbanization, the scale of industrial production and energy consumption has continued to increase. However, as a result, environmental pollution has become more and more serious. Meanwhile, the adverse effects of air pollution have gradually limited sustainable urbanization processes and eco-civilization construction in recent decades [1,2]. In early 2013, China suffered the most severe haze weather since records began [3,4]. Unusual persistent intense air pollution swept the central and eastern regions, attracting widespread concern from society and academia. Therefore, control of atmospheric pollutant discharge and improvements in the urban atmospheric environment quality have become vital goals for China’s current social and economic transformation development [5].

Quantitative analysis of pollutant emissions is fundamental for research on regional atmospheric compound pollution [6,7]. As a key pollutant in fog and haze [8], plenty of literature has investigated the spatial distribution [9], material composition [10,11], sources, and driving factors of PM_2.5_ [12,13]. However, as a pollutant that has an important impact on regional air pollution, NOx is a gas that is not only toxic and harmful, but also has a complex series of effects on the chemical reaction of the troposphere. Due to toxicity, excessive NOx emissions can also harm human health. First of all, short-term exposure of NOx can lead to respiratory diseases, and long-term exposure is causes a greater likelihood of death [14]. In addition, NOx and SO_2_ react with other elements in the atmosphere, resulting in acid rain, and can also lead to respiratory diseases and exacerbate heart disease problems in humans [15]. At the same time, complex chemical reactions may lead to many undesirable phenomena such as photochemical smog in summer, the increase of tropospheric ozone levels in urban areas, and formation of nitrate aerosol (an important oxidant for sulfate formation) [16,17], causing great harm to the ecological environment. NOx is the pollutant which creates the most environmental problems, and as one of the crucial precursors of tropospheric ozone and atmospheric aerosols, an increase of NOx emissions will inevitably lead to further deterioration of haze weather. Hence, the discharge of NOx has provoked concerns from the Government of China (GOC), and the Ministry of Environmental Protection announced the NOx reduction targets during the “12th Five-Year Plan” period, requiring a reduction of 10% in emissions between 2011 and 2015 [18]. Thus, it is urgent to clarify the distribution and evolution of the various regional NOx emissions, and obtain a deeper understanding of the factors affecting NOx emissions reduction from the economic and social points of view.

The research on the total amount of NOx emissions in China is mainly based on bottom-up emissions inventory methods [19]. However, the method of emissions inventory involves great uncertainty due to lack of basic data, local emission factors, and so on [20]. With the rapid development of satellite to earth observation technology, satellite remote sensing as a quantitative analysis method of air pollutant emissions has received greater attention in recent years [21]. The remote sensing observation of NOx is one of the most developed and widely used forms of technology at present [17,22]. In addition, Beirle uses satellite observation to establish a direct link with the approximate surface emissions of NOx as well as NOx concentrations [23]. However, the concentration column of NOx solely reflects the pollution status and cannot directly reflect changes in regional pollutant emissions. At the same time, specific time and resolution of satellite data acquisition will also influence the inversion results. Therefore, regional pollution source censuses and specific monitoring of regional pollutant emission reduction should be undertaken. National environmental statistics have included the NOx factor in the environmental statistics in 2006. The pollution source census carried out in 2007 involved a comprehensive survey on the national NOx emissions coefficient and the discharge of current situation. With the strengthening competence in pollutant emission testing, a breakthrough was achieved in NOx emissions statistics, monitoring and management, laying a foundation for pollutant emissions data received and quantitative analysis.

Meanwhile, the factors affecting NOX emissions are complex. Scholars have begun to use different methods to explore the main influencing factors of NOx emissions in different regions, industries, and sectors. Shi (2014) used the bottom-up method and found that the unbalanced of NOx emissions are mainly due to the gross domestic product (GDP) and industrial structure, while energy consumption is also an important factor influencing NOx emissions in China. Similar methods were also used by Wang and Ohara [24,25] through computing industry emissions inventory, and they found that the development of the steel industry and other heavy industries led to large quantities of NOx emissions. Based on the correlation analysis test, Lamsal found that outdoor ambient NO_2_ concentrations were dependent upon the urban population in different global regions [26]. Taking London as an example, Beevers used different remote sensing data to examine the relationship between the road traffic and NOx emissions [27]. Similar studies have also been conducted in China. Saikawa1 found that as the number of vehicles surged, NOx emissions began to increase rapidly [28]. Based on the IPAT (Environmental Impact = Population × Affluence × Technology) formulation, Shi found that population, affluence, and technology had different potentials for NOx emissions [29]. Importantly, existing works ignore factors such as energy efficiency and energy intensity, and these factors are likely to be crucial factors affecting NOx emissions.

However, studies using rigorous quantitative empirical tools on the influencing factors of NOx remain scarce. At the beginning, most scholars chose traditional multivariate linear regression methods to analyze relevant influencing factors [30]. Later, as the research went further, more and more researchers began to notice the importance of spatial effects in the study of environmental issues. Anselin believes that the use of spatial measurement methods in the study of resources and environmental economics is very necessary [31]. Giacomini and Granger also emphasized the need for considering spatial effects in analyzing the impact of economic development on environmental issues [32]. Moreover, taking the spillover effect of regional economies into account, there may be a spatial effect of provincial pollutant emissions. As a result, the use of traditional multiple regression models like OLS (Ordinary Least Square) and GLS (Generalized Least Squares) may cause large deviations in the estimation results due to the neglect of spatial effects. Thus, in order to make the estimation results more accurate and closer to reality, this paper chooses the spatial econometric model to estimate the influencing factors of NOx.

Recently, more and more experts have begun to apply spatial measurement methods to the research on the influencing factors of environmental pollution. For instance, Marcazzan found there is a significant spatial correlation between the per capita emissions of some important pollutants among different regions [11]. Afterwards, Hosseini and Kaneko revealed that there is a remarkable spatial spillover effect in the regional environment quality, and the impact on the environment in the adjacent areas is obvious [33]. Li et al. began to utilize the spatial models to evaluate the effects of economic development on the environment [34]. In recent studies, Kang et al. found that in some cases the spatial Durbin model has a better explanatory effect, especially in the relationships between economic development and air pollution [35]. At the same time, Hao et al. used the above spatial econometric models (the spatial lag model (SLM), spatial error model (SEM), and spatial Durbin model (SDM)) to explore the impact of some socio-economic factors on urban PM_2.5_ concentrations [36]. Therefore, it is a trend of the current research to incorporate the spatial effect variable into the panel data model for analyzing the pollutant influencing factors.

The main purposes of this paper are to (1) analyze the spatial-temporal distribution characteristics of provincial NOx emissions in China, 2006–2015; and (2) reveal the potential socio-economic driving factors (level of economic development, industrial structure, energy efficiency, urbanization, transportation, and population) of NOx emissions through different spatial econometric models. Compared with previous studies, traditional panel data model and three spatial econometric models (SLM, SEM, and SDM) are applied to this study in order to reduce the bias of estimation results. In terms of explaining variables, we add more social-economic indicators such as energy efficiency and urbanization, as well as the spatial effect variable to obtain a more precise influence process analysis of NOx emissions, as studies related to NOx emissions are still relatively scarce. Meanwhile, a more comprehensive empirical identification of the key influencing factors of NOx emissions is carried out under the conditions of time lag effect, space lag effect, and space-time lag effect. The results will assist the policymakers to set out effective solutions and policies to strengthen NOx emissions reduction in the future plans for air pollution control.

## 2. Data and Empirical Methods

### 2.1. Data Source

The data in this article are all provincial data. The data on NOx emissions for provinces were collected from China Environmental Statistics Yearbook in the period of 2007–2016, and the data on influencing factors including regional gross domestic product (GDP), industrial structure, number of vehicles, energy efficiency, and populations are all from the China Statistical Yearbook from 2007 to 2016. Due to availability and continuity of the data, 31 provinces were considered in this study. In addition, taking the availability of data into account, the Taiwan Province, Hong Kong, and Macao Special Administrative Regions were not included.

### 2.2. Methods

#### 2.2.1. Exploratory Spatial Data Analysis

In order to verify whether the NOx emissions have a non-random spatial distribution and spatial autocorrelation in the occurrence characteristics, this paper performs an exploratory spatial analysis of the selected data [37]. These methods mainly include two forms: the global spatial autocorrelation and local spatial autocorrelation. The former is to explore the distribution characteristics of NOx emissions in the whole study area, while the latter is used to analyze each region of the distribution [38]. This paper uses Moran’s *I* to measure the spatial correlation between adjacent regions in the entire study area: a spatially positive correlation, a spatially negative correlation, or spatial independence [31]. The formula that used to calculate the Moran’s *I* is as follows [39]:

(1) $$\;I=\frac{N\sum_{i}\sum_{j}W_{ij}\left(X_{i}-\bar{X}\right)\left(X_{j}-\bar{X}\right)}{\left(\sum_{i}\sum_{j}W_{ij}\right)\sum_{i}{\left(X_{i}-\bar{X}\right)}^{2}}$$

where *N* is the total number of regions in the study area; $X_{i}$ and $X_{j}$ are the NOx emissions of the regions i and j, respectively; $W_{ij}\;$ is the spatial weight matrix; and $\bar{X}$ is the average of the emissions. Moran’s *I* ranges from −1 to 1, using 0 as the demarcation point for judging positive correlations and negative correlations. When Moran’s *I* is close to 1, it shows that spatial agglomeration is stronger. When it is close to zero, this shows that space has a random distribution, or there is no spatial autocorrelation. A value less than 0 implies a negative autocorrelation.

The local spatial correlation can be analyzed with the Moran scatter plot, implying the distribution characteristics of the selected spatial data in the local spatial field [37]. For a more detailed analysis, the Moran scatterplot can be divided into four quadrants, representing the emissions of NOx on behalf of the province and its neighboring provinces: in quadrant I (HH) both the province itself and the neighboring provinces have higher values; in quadrant III (LL) both the province itself and the neighboring provinces have low NOx emissions. HH and LL denote that there is a positive correlation between provinces. Quadrant II (LH) shows low values surrounded by high values. Quadrant IV (HL) is the opposite to quadrant II. In addition, high and low are defined as relative concepts.

#### 2.2.2. Stochastic Impacts by Regression on Population, Affluence and Technology (STIRPAT) Model

Since the IPAT model is concise and highly representative, it is widely used in analyzing the impact of human factors on environmental changes [40,41]. The influencing factors can be divided into three categories: population, economy, and technology. The model can be shown as follows:

(2)  I=P×A×T

Here, variable *I* is the environmental impact, *P* represents population, *A* means affluence, and *T* is the regional technology level. After the beneficial improvement of different scholars, the IPAT model has some derivative forms, such as the ImPACT model, which decomposes *T* into *T* and *C* [42]. However, both of the two models assume that three influencing factors have the same impact on the environment and are unable to identify which variable’s influence is more significant. Thus, Dietz and Rosa reformed the IPAT model into a random form to overcome this shortcoming [43], and established the STIRPAT model:

(3) $$\;I=\alpha P^{\beta }A^{\gamma }T^{\lambda }\mu$$

where *I*, *P*, *A,* and *T* have the same meaning as in the IPAT model; α  is the constant term; β, γ and λ  are index parameters for each variable, and μ  denotes the random error. The model is a nonlinear model containing multiple independent variables, so it can be converted to logarithmic form:

(4)  lnI=lnα+βlnP+γlnA+λlnT+lnμ

where, ln(.) is a natural logarithm. In this form, β,γ,λ  can be seen as the elastic coefficient, which means when the other influence factors remain unchanged, a 1% change in an impact factor can cause the percentage change in the environment. The STIRPAT model is more flexible in the application process, allowing other explanatory variables to be added [43].

#### 2.2.3. Model Construction

Based on some existing relative studies [44,45], this paper chooses the STIRPAT model for modeling analysis. With NOx emissions representing environment variables, *GDP* (gross domestic product), *IP* (industrial structure), *EI* (energy efficiency), *PC* (the quantity of motor vehicles), *POP* (population), and *UR* (urbanization rate) are introduced into the model, and the extended model (in logarithmic form) is as follows:

(5) $$\begin{array}{c} \;\ln\left(NOx_{it}\right)=\alpha_{0}+\alpha_{1}\ln\left(GDP_{it}\right)+\alpha_{2}\ln\left(IP_{it}\right)+\alpha_{3}\ln\left(EI_{it}\right)+\alpha_{4}\ln\left(PC_{it}\right)\;\\ +\alpha_{5}\ln\left(POP_{it}\right)+\alpha_{6}\ln\left(UR_{it}\right)+\mu_{it} \end{array}$$

where variable *NOx* denotes *NOx* emissions per capita; *GDP* is gross domestic product; *IP* stands for industrial structure (indicated as a proportion of the secondary industry value to the GDP); *EI* represents energy efficiency (expressed as coal consumption of per unit of output); *PC* indicates the number of vehicles (represented as total amount of vehicle at the end of the year). *POP* refers to the population (represented as total population at the end of the year); *UR* is urbanization (the percentage of urban population relative to the total population); and $\mu_{it}$ is the error term. The letter *i* represents regions and *t* indicates time. The definition and the statistical description of the variables used in the study are shown in Table 1.

#### 2.2.4. Spatial Econometric Models

Following Elhorst’s study [46], the spatial econometric model can be divided into many categories based on the relationship between dependent variables and independent variables. The following three ones are widely used in empirical research. They are the spatial lag model (SLM), spatial error model (SEM), and spatial Durbin model (SDM). The first is the SLM, which considers the value of the variable in the space affected by the weighted average of the adjacent variable [47]. It can be written as:

(6)  y=ρWy+Xβ+ε

where y is the dependent variable; X is the exogenous explanatory variable matrix; β is the explanatory variable coefficient; ρ is the spatial regression correlation coefficient; W is the spatially adjacent weight matrix; Wy is the spatially lagged dependent variable, measuring the spatial spillover effect between geographically spatially adjacent regions; and ε is an independently and identically distributed error term with zero mean and variance σ^2^.

During the model setting process, some variables that may be related to dependent variables and have spatial effects are omitted. In some cases, the spatial autocorrelation of the neglected errors will also cause bias in the model estimation [48]. To eliminate this error, the spatial error model (SEM) expression is modified as follows:

(7) $$\;\{\begin{array}{c} y=X\beta +\mu \\ \mu =\lambda W\mu +\varepsilon \end{array}$$

where μ is the random error term vector. μ represents the error term of unit *i*, which is taken to depend on the error terms of neighboring units *j* according to the spatial weights matrix *W* and an idiosyncratic component ε. In addition, ε also is an independently distributed error term with zero mean and variance σ^2^. The parameter λ is the spatial error coefficient of the cross-sectional dependent variable vector, which measures the spatial dependence between the observations of the sample.

The third model uses the spatial Durbin model (SDM), which provides a function with spatially lagged values in both dependent variables and independent variables [49]. It is expressed as:

(8)  y=ρWy+Xβ+WXθ+ε

where θ is a vector of the parameter. This model can then be used to test the hypotheses H_0_: θ=0 and H_0_: θ+λβ=0. The first hypothesis examines whether the spatial Durbin can be simplified to the spatial lag model (SLM), and the second hypothesis whether it can be simplified to the spatial error model (SEM) [46]. Both tests follow a chi-squared distribution with K degrees of freedom. The parameters are the same as in Formulas (6) and (7).

## 3. Spatial and Temporal Characteristics of NOx Emissions

### 3.1. NOx Emissions Reduction Targets and Temporal Evolution

**Figure 1:**
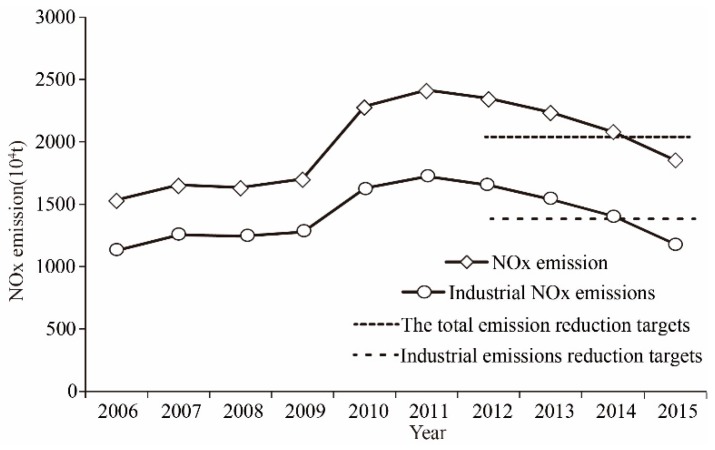
National NOx emissions from 2006 to 2015 and the expected total control targets in 2015.

First, in order to observe the characteristics of NOx emissions over time, a time-varying curve is plotted in Figure 1. It can be seen that the NOx emissions from 2006 to 2008 were relatively stable, followed by a significant upward trend in 2009–2010. Since NOx has been incorporated into the constraints of pollutant emission control during the 12th Five-Year Plan in China, the total emissions of NOx in 2011 began to decline, and finally reduced to 1851.1 million tons in 2015. During this period, the total emission reduction target was 10% [18], and the target was reached.

When it comes to industrial NOx emissions, although the proportion of industrial sources was continually shrinking over these years, it remained at around 70% of the total NOx emissions, and still represent the number one emission source. Industrial emission sources are the key to the management of NOx emission reduction. During the 12th Five-Year Plan period, the government set a target of a 15% reduction of industrial NOx. From the statistical results, the industrial NOx emission level in 2015 was 1.189 billion tons, which was 453.1 million tons less than that in 2010, and the emission reduction task was thus completed. In recent years, due to the continuous urbanization and increasing emissions from vehicles, new challenges and problems with respect to overall emissions reduction have arisen.

Above all, NOx emissions showed first increased, with a subsequent decline. Finally, both emission reduction requirements were met. However, the air pollution situation has not been significantly improved; urban air quality is still terrible, and it is imperative to develop further emission reduction targets.

### 3.2. Spatial Characteristics of NOx Emissions

**Figure 2:**
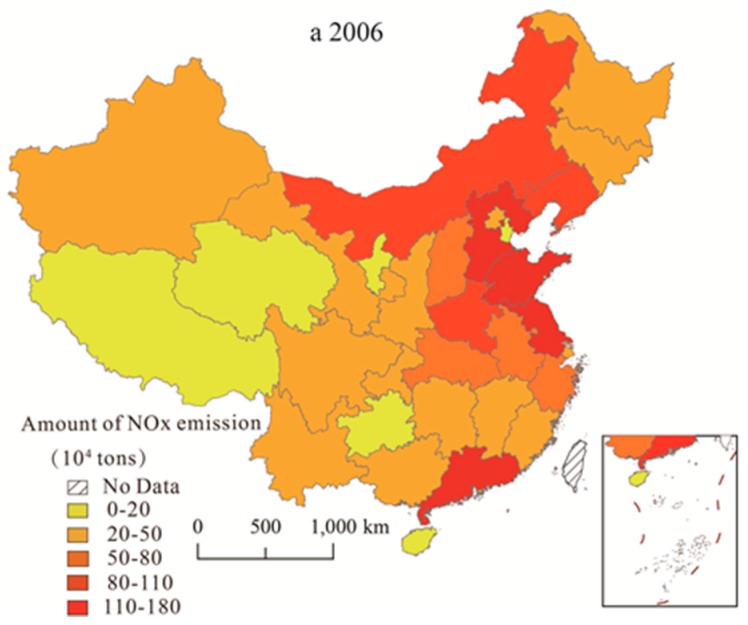

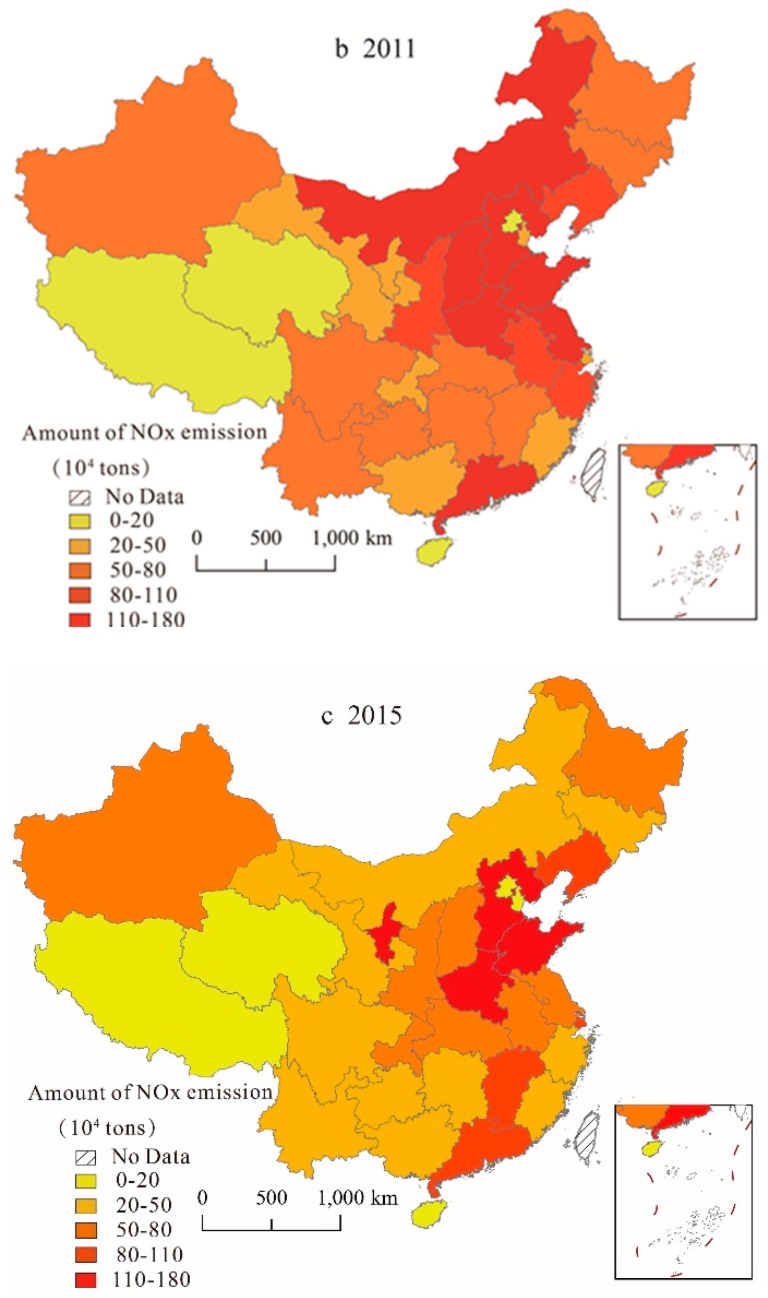
Spatial distribution of provincial NOx emissions in 2006, 2010, and 2015.

To explore the spatial variation of provincial NOx emissions in various provinces and clarify the spatial evolution pattern, ArcGIS 10.2 is used to analyze NOx emissions of 31 provinces from 2006 to 2015. According to the natural fracture point classification [50], five levels are established, ranging from high to low: the low emission zone (0–20×10^4^ tons), the medium-low emission zone (20–50 × 10^4^ tons), the medium emission zone (50–80 × 10^4^ tons), the medium-high emission zone (80–110 × 10^4^ tons), and the high emission zone (110–180 × 10^4^ tons). Then, three analysis sections (2006, 2011, and 2015 correspond to a, b c respectively) are selected to plot the spatial distribution (shows Figure 2).

As a whole, the NOx emissions in most of the regions from 2006 to 2011 were increased, and the number of provinces covered by the medium and high emission levels also increased significantly. After NOx emissions were included in constraint indicators in 2011, levels began to decrease. Specifically, the number of provinces with high emissions rose from four in 2006 to seven in 2011, then dropping to four in 2015. In terms of spatial distribution characteristics, the “hot” points (high emission accumulation areas) are mainly distributed in Hebei, Shandong, and Jiangsu in the eastern and central regions, while the “cold” areas (low-emission accumulation areas) are located in Qinghai and Gansu in the west; these two distribution areas have obvious boundaries and show significant spatial heterogeneity. Furthermore, from a dynamic perspective, the scope of high emission levels began to expand in the surrounding direction from 2006 to 2011, which is consistent with the changes in the NOx concentration column observed by Zhang et al. [51]. Both the latter and present study showed that the original high-emissions areas continuously expanded, and the emission levels in the southeast and northwest regions increased. New high-value areas have continued to appear, showing a clear trend of expansion from the east to the central and western regions. After 2011, the implementation of emission reduction policies led to a certain reduction, and the expansion trend was curbed.

In general, the provincial NOx emissions have prominent spatial agglomeration effects and spatial heterogeneity. These high-emission agglomeration areas are vital areas for future pollution control, and effective treatment plans must be developed to prevent the emergence of new high-emission zones.

## 4. Estimation Results and Discussion

### 4.1. Estimation Results of Traditional Panel Data Model

(1) Panel unit root test

In order to avoid the occurrence of spurious regressions and ensure the validity of the estimation results, it is necessary to test the stability of the data. This paper used the three kinds of unit root test methods: Adjusted Dickey–Fuller (ADF) inspection, and the Phillips-Perron (PP) and Levin–Lin–Chu (LLC) tests [52]. The results of the unit root test for each variable are shown in Table 2, which shows that all variables are stable at the level and are significant at the 5% level.

(2) Pedroni co-integration test

The Pedroni co-integration test was utilized to find out whether a long-term relationship existed between influence factors and NOx emissions. Under the condition of the small sample (that is, a time span <20 years), the panels of ADF statistics and Group ADF statistics are more effective [53]. Table 3 shows the co-integration test results that there is a co-integration relationship between each variable and NOx emissions at a 5% significant level.

(3) Hausman test

The Hausman test is used to determine if we should use the fixed model or the random model. When the Hausman test results’ *p*-value is greater than 10%, we should accept the null hypothesis of the “set up a random model,” or should choose the fixed model. According to the result (*P* = 0), a fixed model should be selected to analyze the affecting factors of NOx emissions.

### 4.2. Spatial Autocorrelation Test

According to the study of Han et al. on provincial PM_2.5_ [54], China’s air pollution shows a significant spatial correlation, meaning that pollution in an area is not only affected by its own characteristics but may also be affected by the air quality of neighboring regions. In this case, the use of traditional regression models such as OLS and GLS will lead to biased estimate due to neglect of spatial effects. To address this problem, firstly, we need to determine whether there is spatial correlation between the NOx emissions of different provinces.

This paper takes advantage of Geoda to explore spatial correlation, and then calculates Moran’s *I* in 2006–2015, with E(*I*) as the expected value and *p*-Values respecting the level of significance. The results are shown in Table 4. The Moran’s *I* values are all within the range of (0.156, 0.351), and the Monte Carlo test is significant at the 0.05 level, indicating that the provincial NOx emissions show a clustering distribution, and there is a remarkable spatial correlation relationship between emissions in neighboring provinces.

**Figure 3:**
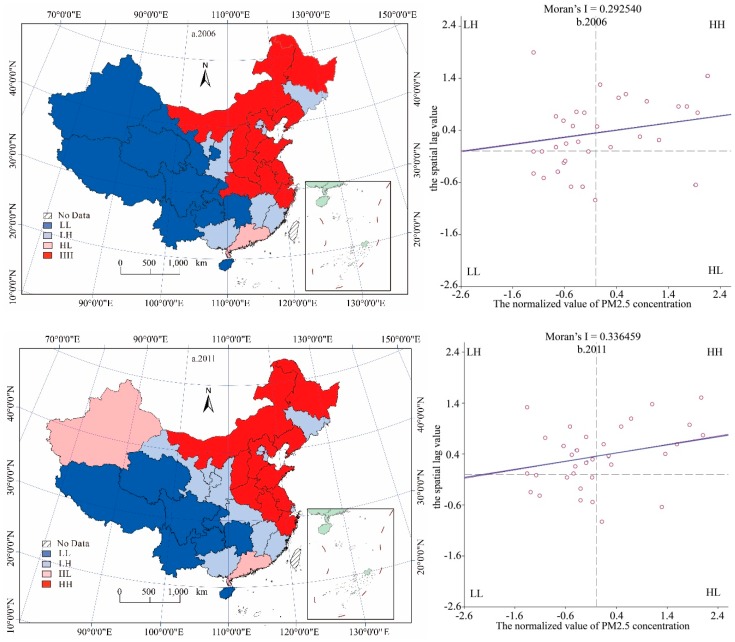

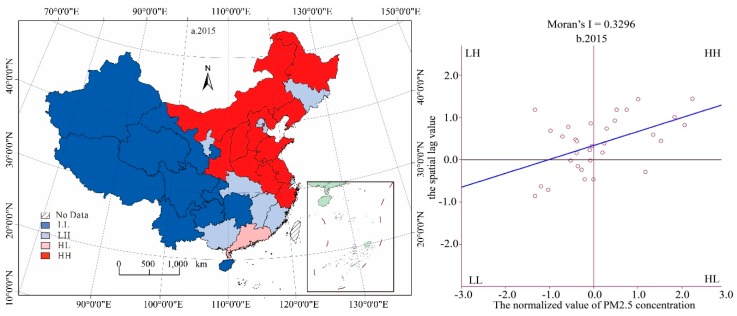
Moran scatter plot of per capital NOx emissions in 2006, 2011, and 2015. a, b correspond to the left part and the right part respectively. The right part shows quadrant distributions NOx emissions, and the left part shows the corresponding spatial patterns.

Relatively speaking, the local spatial correlation can be expressed by the Moran scatter plot, where the abscissa is the observed value of each provincial variable, and the ordinate is the average of observations in an area and its surrounding area [55]. The right section of Figure 3 shows the quadrant distributions of NOx emissions, while the left section displays the spatial representation of the Moran scatter plot. The four quadrants in figure represent four types of spatial autocorrelation, respectively. NOx emissions have obvious local spatial agglomeration characteristics. HH and LL represent agglomeration areas, classified as HH-type provinces that tend to be distributed in the middle east region of China (such as Hebei, Shandong, and Jiangsu), and LL-type provinces that are mainly concentrated in western regions like Qinghai, and Tibet, and southwest of Sichuan, Yunnan and other provinces. The LH classification was mainly distributed in the transition zone between the HH and LL areas. In 2006, only Guangdong belonged to the HL area, and later in 2011 Xinjiang also, where due to the increase in emissions in some areas some LL classifications then transformed into LH classifications. Then, the provincial NOx emissions of spatial agglomeration had a tendency to reduce. However, in the 2015 the situation again became similar to that in 2006, where the emissions decreased and the spatial agglomeration was reflected once again. In conclusion, we can see that there is a strong spatial correlation between provincial NOx emissions, so when we use a panel analysis, we must take the spatial factors in to consideration and then choose the appropriate spatial panel model.

### 4.3. Spatial Econometric Regression Results

For the purpose of detecting which model is the most suitable for this study, the non-spatial panel model for regression estimation is chosen first, and the results are shown in Table 5. Then, the LR tests is performed to detect the significance of the fixed effects and seek which fixed effect is most suitable for this study—the time-period fixed effect or the spatial fixed effect. The results demonstrate that null hypothesis of time period fixed effects is rejected, but the null hypothesis of spatial fixed effects is accepted. Therefore, the panel data model with time-period fixed effects is chosen as the best model.

According to the results of LM test, it can be seen that there is no spatial error. Thus, the SEM is excluded first. Furthermore, according to the Wald test and the significance of the estimated results, the SDM is more suitable for this study than the SLM. At the same time, considering the LR test comprehensively, the SDM with time-period fixed effect is the best fitted one for the estimation. The estimated results are shown in Table 6.

From Table 6, we can see there are big differences in the effect of the explanatory variables for NOx emissions. Based on the estimation results of SDM, the six explanatory variables all have a positive effect on NOx emissions, but this is not accurate enough to use the coefficients for impact analysis [56]. Thus, we can only use the parameters to explain why the explanatory variables have a positive impact on NOx emissions.

The first variable is energy efficiency. As we are aware, fossil fuel combustion, especially of coal and petroleum, has a significant impact on NOx emissions. On one hand, prior studies have shown that 70% of NOx emissions come from coal combustion [57]. On the other hand, the energy structure in China is “coal-rich, oil-poor, lack of natural gas”, and under the current process of industrialization and urbanization, this means that the “coal-based” energy structure will exist for a long time in China. Apart from the large amount of coal consumption, due to the backward production technology, coal displays incomplete combustion and low efficiency [58]. This phenomenon exacerbates the quantities of pollutants generated into the atmosphere, and makes air quality worse.

Another important source of NOx emissions is the continuous development of the secondary industry, which means the increase in the proportion of secondary industrial output will also lead to increase in NOx emissions. According to statistics, nearly 70% of fossil energy is used by the secondary industry, meaning that the development of the secondary industry will cause greater use of fossil fuel. For example, the power industry and heating industry all require large quantities of energy sources. Furthermore, other industries like the cement industry and steel industry will also produce large quantities of NOx emissions in the production process. In general, the combustion of fossil fuels caused by the development of secondary industries is the main reason for the increase in NOx emissions. The production process of the cement industry, steel industry, and other industries are also a key source of NOx emissions.

Similarly, the urbanization rate also has a positive and significant effect on NOx emissions. China is in a stage of rapid development, and sustainable development in industrialization and urbanization is in progress. However, development with high energy consumption and a high pollution pattern is still the dominating phenomenon [35]. From the statistical results, more and more NOx emissions come from activities of daily life, especially in urban areas. As a result, urbanization could lead to a more rapid expansion of air pollution in large cities.

As we can see from the results of the model estimation, the coefficient of the population is positive, suggesting that the population gathering tends to increase NOx emissions. In the process of rapid urbanization in China, the increase in the population will produce greater resource consumption and waste emissions, and place greater pressure on the environment.

The effect of the GDP on NOx emissions is worth further discussion; we cannot simply say that the impact is positive or negative firsthand. As we can see from the results of the model estimation the coefficient of the population is positive, which suggests that greater GDP tends to increase NOx emission. At this stage regions are still in extensive mode of economic development accompanied by high energy consumption and high pollution. However, Dinda has proven that there is an inverted-U-shaped relationship between different pollutants and per capita income [59]. Thus, GDP growth has a different degree of influence on the air quality at different stages of development [60].

Although the estimation result of the vehicular indicator does not pass the significance test, we also found that the effect of increasing number of vehicles on NOx emissions was of great concern to the public [61]. Vehicular exhaust has an important impact on the atmospheric environment. At present, according to statistics of China Environment Statistical Yearbook, vehicular gas has become the second largest source of NOx emissions. In the past few years there has been extremely quick growth in the number of vehicles, and this trend of rapid increase in urban areas is likely to continue in the future. Eventually, air pollution caused by vehicular gas in cities will become the most important environmental challenge in China, and thus we will need to place greater focus on the control of vehicles.

### 4.4. Direct and Indirect Effects

According to the earlier study [55], the emissions of one region not only depend on its own factors but are also affected by the adjacent areas. Thus, we use the estimation results of direct and indirect effects to distinguish the degree of impact of explanatory variables and spatial spillover effects (shown in Table 7).

We can see from the Table 7, from the estimation results of direct effects only the modulus of the vehicular indicator does not pass the significance test. The coefficient of GDP is 0.2211, which means that economic development in the research areas causes an aggravation of NOx pollution in the same areas. At the same time, it is seen that the selected regions are still in an extensive mode of economic development. In order to continue the expansion of economy, these regions must be transformed into an environment-friendly mode. The estimate of industrial structure is 0.8895, which shows that increasing the proportion of the secondary industry can lead to NOx pollution. The remaining three indicators also show significant positive effects on their own regions, and they all can make NOx pollution worse. Therefore, it is necessary to give greater attention to these impact factors.

When it comes to indirect effects, only three indicators passed the significant test. The estimated GDP is 0.8917, indicating that increased GDP exacerbates adjacent NOx emissions, a phenomenon which is caused by the spillover effect. The indirect effect of energy efficiency is estimated to be 0.4486, indicating that the increase of coal consumption of per unit of output in one region will increase the pollution discharge in the nearby area. All of these factors require us to pay attention to regional cooperation in the process of atmospheric environmental control. The indirect effect of the vehicular indicator is estimated as −0.4716, which shows that the increase in vehicular emissions may reduce the pollution of the adjacent areas. This is because automobile exhaust has a greater impact on the region from which it is emitted. At the same time, the increase in the number of vehicles in one place may affect the number in adjacent areas.

### 4.5. Policy Implications

In accordance with the previous research, we can give some constructive policy recommendations to promote a further reduction of NOx emissions in China. Firstly, NOx pollution is a serious threat to human health and daily life. Therefore, policymakers need to realize the urgency of pollutant emission control. Of course, economic development is prerequisite, but we reject extensive economic growth and must abandon the development concept of “pollute first and then control”. Broadly speaking, regional development has a significant impact on NOx emissions locally and in surrounding areas. Therefore, policymakers should pay more attention to the situation in the region and the surrounding areas at the same time and place greater focus on regional cooperation in order to achieve joint prevention and control of pollutant emissions. Finally, according to the analysis of the spatial panel model, the influencing factors have different degrees of effects on NOx emissions, and thus effective policy recommendations are given.

For energy efficiency, while maintaining a certain amount of coal consumption at this stage, we should improve energy efficiency and reduce the amounts of NOx produced. We should encourage the adoption of clean coal technology, rebuild thermal power plants, update coal-fired industrial boilers, and increase combustion efficiency to realize the improvement of energy efficiency and clean use of coal energy. It must be admitted that increasing the share of clean energy through means such as wind power, solar power, hydropower, and nuclear energy is also a very effective tool. What is more, we also need to curb excessive coal consumption through tax policy and support the development of clean energy through government subsidies.

For the industrial structure, as we mentioned before, secondary industry development led to NOx emissions of almost 70%. Hence, reducing industrial pollution is the most effective measure to improve air quality. Most significantly, we should optimize the industrial structure and focus on the development of the tertiary industry, namely low power consumption and small resource-dependent industries. In the current context of rapid economic development, industrial restructuring is a long and arduous task, especially in districts receiving transferred industries. High emission project review standards need to be created, while strengthening the standards of Environmental Impact Assessment (EIA) to control the establishment and development of highly polluting industries.

Furthermore, China’s urbanization rate reached 57.35% in 2016, and this proportion is still increasing. Hence, China should vigorously promote low-carbon cities, emphasize ecological civilization, use energy and other resources economically, and promote construction of green cities and low-carbon lifestyles in order to reduce the damage to the environment caused by rapid urban development.

Automotive exhaust has become the second largest source of NOx emissions. The rapid increase in the number of motor vehicles not only increases the emission of atmospheric pollutants but also leads to denser traffic [62]. Traffic jams contribute to pollutant emissions. In order to alleviate this situation, policymakers need to place emphasis on the growth rate of motor vehicles in megacities. More significant and feasible measures to control the number of vehicles include restricting the supply of the license plate numbers, and odd license plate restriction rules can be performed simultaneously. However, in the long run, the rapid growth in the number of private cars is difficult to control. Thus, the government should not simply rely on the fuel consumption tax adjustment, but also should encourage the development of clean energy and develop the low-emission cars known as “Green” vehicles [63].

## 5. Conclusions

To some extent, according to the experience of the developed countries, the current environmental issue is an inevitable stage to be experienced in the process of China’s economic development, and it may take China some time to manage and improve the situation. In this context, we need more intensive study on the relationship between China’s NOx emissions and the driving socio-economic factors. Based on the provincial panel data set in the period of 2006–2015, this paper used an extended STIRPAT model and spatial econometric models to investigate the socioeconomic influential factors of NOx emissions and their spatiotemporal patterns. Firstly, provincial NOx emissions in China increased in volatility as time went on, and then decreased in 2011 after the introduction of restrictive policies. At the same time, due to China’s huge land area and imbalanced regional development, different regions have different NOx emission patterns and reduction discrepancies.

In terms of the spatial correlation test, there are spatial correlations between different provinces. This means that the provincial NOx emission changes not only affected the provinces themselves, but also affected neighboring regions. Thus, we chose spatial econometric methods to explore the influencing factors of NOx emissions. From the statistics of LM test and Wald test, the Durbin model with time-period effects fixed is the most suitable method for the study. Lastly, spatial panel econometric model analysis results showed that the explanatory variables all have a positive effect on NOx emissions except for vehicles (a variable which did not pass the significant test). From the estimates of direct and indirect effects we can see that the indicators also show significant positive effects on the own areas, i.e., they all can make air pollution even worse, with the exception of the vehicular indicator, which did not pass the test. Simultaneously, only three indicators passed the significance test, among which the increase of GDP and energy efficiency exacerbated adjacent NOx pollution.

In addition, it is worth noting that many scholars have focused on the issue of multinational pollution transfer and its impact [64] in recent years. From the existing literature research, the impact of multinational pollution transfer is mainly estimated and simulated through international trade data [65]. Of course, such research is of great significance to air pollution control. In a sense, the effects of atmospheric transport and international trade on air pollution are key to explaining the spatial effects of pollutant emissions [66,67]. Therefore, from a global perspective, we still need to focus upon and emphasize the regional and transboundary transfer of NOx emissions. In subsequent studies, when we obtain more detailed cross-country data on NOx emissions, we will further explore spatial diffusion and impact effects based on the research ideas and analysis framework in this study.

## Figures and Tables

**Table 1:** Definition and the statistical description of all relevant variables used in the study.

Variables	Definition	Units of Measurement	Mean	Std.	Skewness	Min	Max
*NOx*	NOx emissions	Ton	63.378	43.360	0.800	0.036	180.100
*GDP*	GDP	10^8^ Yuan	15,444.160	13,743.211	1.763	290.760	72,812.550
*EI*	energy efficiency	Ton per 10^4^ yuan	1.096	0.584	1.402	0.298	3.860
*IP*	industrial structure	Percent	39.520	9.723	−1.637	6.808	53.036
*UR*	urbanization	Percent	51.433	14.611	0.793	21.053	89.607
*PC*	number of vehicles	10^4^ units	295.274	277.172	1.907	9.820	1510.810
*POP*	population	10^4^ units	4305.997	2719.806	0.581	285.000	10,849.000

**Table 2:** Results of the panel unit root test.

Title Difference	Variable	ADF	PP	LLC
Level	*lnNOx*	161.582 ***	177.616 ***	−12.355 ***
	*lnGDP*	129.093 ***	159.347 ***	−11.102 ***
	*lnIP*	162.165 ***	162.742 ***	−15.041 ***
	*lnEI*	105.052 ***	88.513 **	−8.442 ***
	*lnPC*	150.982 ***	168.818 ***	−12.118 ***
	*lnPOP*	145.757 ***	152.984 ***	−12.836 ***
	*lnUR*	157.066 ***	176.055 ***	−11.873 ***

Notes: ** Significance at 5% level. *** Significance at 1% level. ADF: Adjusted Dickey–Fuller; LLC: Levin–Lin–Chu.

**Table 3:** Results for co-integration between NOx emissions and their influencing factors.

Variable	*lnGDP*	*lnIP*	*lnEI*	*lnPC*	*lnPOP*	*lnUR*
Panel PP statistics	−5.934 ***	−19.161 ***	−4.723 ***	−10.839 ***	−13.458 ***	−7.498 ***
Panel ADF statistics	−9.942 ***	−12.253 ***	−4.878 ***	−9.226 ***	−8.928 ***	−7.318 ***
Group PP statistics	−5.317 ***	−6.136 ***	−3.227 ***	−6.346 ***	−3.563 ***	−3.655 ***
Group ADF statistics	−3.620 ***	−4.091 ***	−2.226 **	−4.307 ***	−2.569 ***	−3.375 ***

Notes: ** Significance at 5% level. *** Significance at 1% level.

**Table 4:** Global Moran’s *I* estimate based on the Monte Carlo test.

Years	Moran’s *I*	Sd.	*p*-Value
2006	0.2925	0.0117	0.0029
2007	0.1565	0.0117	0.0836
2008	0.2125	0.0116	0.0245
2009	0.2159	0.0118	0.0233
2010	0.3513	0.0116	0.0004
2011	0.3365	0.0117	0.0007
2012	0.3311	0.0117	0.0008
2013	0.3185	0.0117	0.0013
2014	0.3307	0.0107	0.0040
2015	0.3296	0.0110	0.0040

**Table 5:** Estimation results of the traditional panel data model.

Determinants	Pooled OLS	Spatial Fixed Effects	Time-Period Fixed Effects	Spatial and Time-Period Fixed Effects
*lnGDP*	0.815191 ***	0.001386	0.611203 ***	−0.010994
*lnIP*	1.549114 ***	0.772261 ***	1.023624 ***	0.765081 ***
*lnEI*	0.152898	0.797270 ***	0.754281 ***	0.801505 ***
*lnPC*	0.342133 **	0.082009	0.201438 **	0.099901
*lnPOP*	−0.574377 ***	0.874274 ***	0.081147	0.872824 ***
*lnUR*	−1.656666 ***	1.003839 **	0.112472	1.007261 **
R^2^	0.750777	0.8549944	0.81510	0.857801
DW	1.741779	2.360017	2.088705	2.384704

Notes: ** Significance at 5% level. *** Significance at 1% level.

**Table 6:** Estimation results of time-period fixed panel data model.

Variables	SEM	SLM	SDM
*lnGDP*	0.261 *	0.219	0.252 *
*lnIP*	0.822 ***	0.842 ***	0.887 ***
*lnEI*	0.978 ***	0.963 ***	0.957 ***
*lnPC*	0.032	0.081	0.063
*lnPOP*	0.660 ***	0.643	0.618 ***
*lnUR*	1.021 ***	0.940 ***	0.666 ***
W×GDP	-	-	1.048 ***
W×IP	-	-	0.016
W×EI	-	-	0.664 ***
W×PC	-	-	−0.517 ***
W×POP	-	-	−0.333
W×UR	-	-	0.455 *
$\sigma^{2}$	0.208	0.212	0.197
$R^{2}$	0.834	0.836	0.848

Notes: * Significance at 10% level. *** Significance at 1% level. SLM: spatial lag model; SEM: spatial error model; SDM: spatial Durbin model.

**Table 7:** Estimation results of direct and indirect effects.

Variables	Direct	Indirect	Total
*lnGDP*	0.221141 *	0.891747 **	1.11288 ***
*lnIP*	0.889481 ***	−0.103813	0.785668 **
*lnEI*	0.941006 ***	0.448607 **	1.389613 ***
*lnPC*	0.0770136	−0.471638 **	−0.394625 **
*lnPOP*	0.634066 ***	−0.385499	0.248567
*lnUR*	0.656849 **	0.319679	0.976528 *

Notes: * Significance at 10% level. ** Significance at 5% level. *** Significance at 1% level.

## References

[1] Qin Y., Zhu H. (2018). Run away? Air pollution and emigration interests in China. J. Popul. Econ..

[2] Chen R.J., Wang X., Meng X., Hua J., Zhou Z.J., Chen B.H., Kan H.D. (2013). Communicating air pollution-related health risks to the public: An application of the air quality health index in Shanghai, China. Environ. Int..

[3] Wang Z.B., Fang C.L. (2016). Spatial-temporal characteristics and determinants of PM_2.5_ in the Bohai Rim Urban Agglomeration. Chemosphere.

[4] Tian S.L., Pan Y.P., Liu Z.R., Wen T.X., Wang Y.S. (2014). Size-resolved aerosol chemical analysis of extreme haze pollution events during early 2013 in urban Beijing, China. J. Hazard. Mater.

[5] Huang W.Q., Fan H.B., Qiu Y.F., Cheng Z.Y., Xu P.R., Qian Y. (2016). Causation mechanism analysis for haze pollution related to vehicle emission in Guangzhou, China by employing the fault tree approach. Chemosphere.

[6] Wang S.J., Fang C.L., Guan X.L., Pang B., Ma H.T. (2014). Urbanization, energy consumption, and carbon dioxide emissions in China: A panel data analysis of China’s provinces. Appl. Energy.

[7] Fang C., Liu H., Li G., Sun D., Miao Z. (2015). Estimating the impact of urbanization on air quality in China using spatial regression models. Sustainability.

[8] Zhang X., Zhang X., Chen X. (2017). Valuing air quality using happiness data: The case of China. Ecol. Econ..

[9] Han L.J., Zhou W.Q., Li W.F. (2014). Impact of urbanization level on urban air quality: A case of fine particles (PM_2.5_) in Chinese cities. Environ. Pollut..

[10] Yao X.H., Chan C.K., Fang M., Cadle S., Chan T., Mulawa P., He K.B., Ye B.M. (2002). The water-soluble ionic composition of PM_2.5_ in Shanghai and Beijing, China. Atmos. Environ..

[11] Marcazzan G.M., Vaccaro S., Valli G., Vecchi R. (2001). Characterisation of PM_10_ and PM_2.5_ particulate matter in the ambient air of Milan (Italy). Atmos. Environ..

[12] Guan D.B., Su X., Zhang Q., Peters G.P., Liu Z., Lei Y., He K.B. (2014). The socioeconomic drivers of China’s primary PM_2.5_ emissions. Environ. Res. Lett..

[13] Xu B., Lin B. (2016). Regional differences of pollution emissions in China: Contributing factors and mitigation strategies. J. Clean Prod..

[14] Mauzerall D.L., Sultan B., Kim N., Bradford D.F. (2005). NOx emissions from large point sources: Variability in ozone production, resulting health damages and economic costs. Atmos. Environ..

[15] Boningari T., Smirniotis P.G. (2016). Impact of nitrogen oxides on the environment and human health: Mn-based materials for the NOx abatement. Curr. Opin. Chem. Eng..

[16] Weschler C.J. (2006). Ozone’s impact on public health: Contributions from indoor exposures to ozone and products of ozone-initiated chemistry. Environ. Health Perspect..

[17] Lin J.T., McElroy M.B., Boersma K.F. (2010). Constraint of anthropogenic NOx emissions in China from different sectors: A new methodology using multiple satellite retrievals. Atmos. Chem. Phys..

[18] Wang S.X., Xing J., Zhao B., Jang C.R., Hao J.M. (2014). Effectiveness of national air pollution control policies on the air quality in metropolitan areas of China. J. Environ. Sci..

[19] Huang C., Chen C.H., Li L., Cheng Z., Wang H.L., Huang H.Y., Streets D.J., Zhang G.F., Chen Y.R. (2011). Emission inventory of anthropogenic air pollutants and VOC species in the Yangtze River Delta region, China. Atmos. Chem. Phys..

[20] Zhang Q., Streets D.G., Carmichael G.R., He K.B., Huo H., Kannari A., Klimont Z., Park I.S., Reddy S., Fu J.S. (2009). Asian emissions in 2006 for the NASA INTEX-B mission. Atmos. Chem. Phys..

[21] Martin R.V. (2008). Satellite remote sensing of surface air quality. Atmos. Environ..

[22] Richter A., Burrows J.P., Nüß H., Granier C., Niemeier U. (2005). Increase in tropospheric nitrogen dioxide over China observed from space. Nature.

[23] Beirle S., Platt U., Wenig M., Wagner T. (2003). Weekly cycle of NO_2_ by GOME measurements: A signature of anthropogenic sources. Atmos. Chem. Phys..

[24] Wang K., Tian H., Hua S., Zhu C., Gao J., Xue Y., Hao J.M., Wang Y., Zhou J. (2016). A comprehensive emission inventory of multiple air pollutants from iron and steel industry in China: Temporal trends and spatial variation characteristics. Sci. Total Environ..

[25] Ohara T., Akimoto H., Kurokawa J., Horii N., Yamaji K., Yan X., Hayasaka T. (2007). An Asian emission inventory of anthropogenic emission sources for the period 1980–2020. Atmos. Chem. Phys..

[26] Lamsal L.N., Martin R.V., Parrish D.D., Krotkov N.A. (2013). Scaling relationship for NO_2_ pollution and urban population size: A satellite perspective. Environ. Sci. Technol..

[27] Beevers S.D., Westmoreland E., de Jong M.C., Williams M.L., Carslaw D.C. (2012). Trends in NOx and NO_2_ emissions from road traffic in Great Britain. Atmos. Environ..

[28] Saikawa E., Kurokawa J.I., Takigawa M., Borken-Kleefeld J., Mauzerall D.L., Horowitz L.W., Ohara T. (2011). The impact of China’s vehicle emissions on regional air quality in 2000 and 2020: A scenario analysis. Atmos. Chem. Phys..

[29] Shi Y., Xia Y.F., Lu B.H., Liu N., Zhang L., Li S.J., Li W. (2014). Emission inventory and trends of NOx for China, 2000–2020. J. Zhejiang Univ. Sci. A.

[30] Ericsson E. (2001). Independent driving pattern factors and their influence on fuel-use and exhaust emission factors. Transp. Environ..

[31] Anselin L. (2001). Spatial effects in econometric practice in environmental and resource economics. Am. J. Agric. Econ..

[32] Giacomini R., Granger C.W.J. (2004). Aggregation of space-time processes. J. Econom..

[33] Hosseini H.M., Kaneko S. (2013). Can environmental quality spread through institutions?. Energy Policy.

[34] Li Q., Song J.P., Wang E.R., Hu H., Zhang J.H., Wang Y.Y. (2014). Economic growth and pollutant emissions in China: A spatial econometric analysis. Stoch. Environ. Res. Risk. Assess..

[35] Kang Y.Q., Zhao T., Yang Y.Y. (2016). Environmental Kuznets curve for CO_2_ emissions in China: A spatial panel data approach. Ecol. Indic..

[36] Hao Y., Liu Y.M. (2016). The influential factors of urban PM_2.5_ concentrations in China: A spatial econometric analysis. J. Clean. Prod..

[37] Rey S.J. (2001). Spatial empirics for economic growth and convergence. Geogr. Anal..

[38] Geng Y., Wang M.L., Sarkis J., Xue B., Zhang L., Fujita T., Yu X.M., Ren W.X., Zhang L.M., Dong H.J. (2014). Spatial-temporal patterns and driving factors for industrial wastewater emission in China. J. Clean. Prod..

[39] Long R.Y., Shao T.X., Chen H. (2015). Spatial econometric analysis of China’s province-level industrial carbon productivity and its influencing factors. Appl. Energy.

[40] Raskin P.D. (1995). Methods for estimating the population contribution to environmental change. Ecol. Econ..

[41] York R., Rosa E.A., Dietz T. (2002). Bridging environmental science with environmental policy: Plasticity of population, affluence, and technology. Soc. Sci. Q..

[42] Waggoner P.E., Ausubel J.H. (2002). A framework for sustainability science: A renovated IPAT identity. Proc. Natl. Acad. Sci. USA.

[43] Dietz T., Rosa E.A. (1994). Rethinking the environmental impacts of population, affluence and technology. Hum. Ecol. Rev..

[44] Zilio M., Recalde M. (2011). GDP and environment pressure: The role of energy in Latin America and the Caribbean. Energy Policy.

[45] Wang L.P., Guan J., Zhang J.D. (2010). Environmental pollution and economic growth in China: A dynamic spatial panel data model. Sci. Geograp. Sin..

[46] Elhorst J.P. (2014). Matlab software for spatial panels. Int. Reg. Sci. Rev..

[47] Zhao X., Burnett J.W., Fletcher J.J. (2014). Spatial analysis of China province-level CO_2_ emission intensity. Renew. Sustain. Energy Rev..

[48] Maddison D. (2006). Environmental Kuznets curves: A spatial econometric approach. J. Environ. Econ. Manag..

[49] Yang Q., Liu H.J. (2012). Regional difference decomposition and influence factors of China’s carbon dioxide emissions. Quan. Tech. Econ..

[50] Jiang B. (2013). Head/tail breaks: A new classification scheme for data with a heavy-tailed distribution. Prof. Geogr..

[51] Zhang Q., Geng G.N., Wang S.W., Andreas R., He K.B. (2012). Satellite remote sensing of changes in NOx emissions over China: 1996–2010. Chin. Sci. Bull..

[52] Wang S.J., Zhou C.S., Li G.D., Feng K.S. (2016). CO_2_, economic growth, and energy consumption in China’s provinces: Investigating the spatiotemporal and econometric characteristics of China’s CO_2_ emissions. Ecol. Indic..

[53] Wang S.J., Li Q.Y., Fang C.L., Zhou C.S. (2016). The relationship between economic growth, energy consumption, and CO_2_ emissions: Empirical evidence from China. Sci. Total Environ..

[54] Han L.J., Zhou W.Q., Li W.F. (2014). City as a major source area of fine particulate (PM_2.5_) in China. Environ. Pollut..

[55] Wang S.J., Fang C.L., Wang Y. (2016). Spatiotemporal variations of energy-related CO_2_ emissions in China and its influencing factors: An empirical analysis based on provincial panel data. Renew. Sustain. Energy Rev..

[56] Xiang K., Song D. (2015). Spatial analysis of China’s PM_2.5_ pollution at the provincial level. China Pop. Res. Environ..

[57] Shi Y.L., Cui S.H., Xu S., Lin J.Y., Huang W. (2014). Nitrogen oxide emission in energy consumption in China from a consumption-based perspective. Acta Sci. Circum..

[58] Yang G.F., Li W.L., Wang J.L., Zhang D.Q. (2016). A comparative study on the influential factors of China’s provincial energy intensity. Energy Policy.

[59] Dinda S. (2004). Environmental Kuznets curve hypothesis: A survey. Ecol. Econ..

[60] Li Y.W., Xu Z.M., Wang Y., Jiao W.X. (2005). Study on the Environmental Kuznets Curve. China Pop. Res. Environ..

[61] Xue W.F., Fu F., Wang J.N., Tang G.Q., Lei Y., Yang J.T., Wang Y.S. (2014). Numerical study on the characteristics of regional transport of PM_2.5_ in China. China Environ. Sci..

[62] Zhou Y., Wu Y., Yang L., Fu L.X., He K., Wang S.X., Hao J.M., Chen J.C., Li C. (2010). The impact of transportation control measures on emission reductions during the 2008 Olympic Games in Beijing, China. Atmos. Environ..

[63] Tielert T., Killat M., Hartenstein H., Luz R., Hausberger S., Benz T. The impact of traffic-light-to-vehicle communication on fuel consumption and emissions. Proceedings of the IEEE Internet of Things.

[64] Hao Y., Peng H., Temulun T., Liu L.Q., Mao J., Lu Z.N., Chen H. (2018). How harmful is air pollution to economic development? New evidence from PM_2.5_ concentrations of Chinese cities. J. Clean. Prod..

[65] Noailly J., Ryfisch D. (2015). Multinational firms and the internationalization of green R&D: A review of the evidence and policy implications. Energy Policy.

[66] Ding L., Liu C., Chen K.L., Huang Y.L., Diao B.D. (2017). Atmospheric pollution reduction effect and regional predicament: An empirical analysis based on the Chinese provincial NOx emissions. J. Environ. Manag..

[67] Zhao H., Li X., Zhang Q., Jiang X., Lin J., Peters G.P., Zhang L. (2017). Effects of atmospheric transport and trade on air pollution mortality in China. Atmos. Chem. Phys..

